# Supplementary material for: The effects of COVID-19 mitigation measures on physical activity (PA) participation among adults in Rwanda: An online cross-sectional survey
Source: PLoS One. 2023 Nov 9;18(11):e0293231. doi: 10.1371/journal.pone.0293231 (PMC10635554; doi:10.1371/journal.pone.0293231)
Supplement: Attachment — Submitted filename: PONE-D-22-31575R1_Dr. SH.pdf [file pone.0293231.s002.pdf]

Thank you for your submitted manuscript entitled, PONE-D-23-13290 ''The Effects of COVID-19 Mitigation Measures on Physical Activity Participation among Adults in Rwanda: an Online Cross-sectional Survey''.

ABSTRACT:

- In the Methods provides more information about the PA and sedentary time assessment used in the study.
- How you determined the ''self-administered questionnaire''.
- Could be a relevant conclusion for the present study. However, you note in the conclusion that ''COVID-mitigation measures have significantly reduced PA participation and increased sedentary time''.. This statement is not a new discovery. Although, what is the new discovery?

INTRODUCTION:

- Line 97-108: The development of the introduction needs to be more hypotheses driven and develop the questions leading up to the section in the methods section. In this section, there is no new information in the paper this is reported in many published papers, it is necessary to remake and reinforce this part by other evidence, information and bibliographic references. Further, the part on hypothesis/-es development needs revision. A more conclusive use of appropriate literature can help here to clearly state the purpose of the study in order to develop hypotheses (or null hypotheses, whichever way is preferred). In turn, this will serve as the paper's framework as it will define the importance of findings, i.e. which results are being presented. In its present form, the paper states one quite unspecific hypothesis, therefore, results presented seem random which also influences the discussion.

METHOD

- The procedure is rigorous and well described and the statistical analysis is correct.

RESULTS:

- What about the measure of data reliability in the study?
- The tables are poorly presented, need formatting. Take time to check all formatting and make sure that all of the tables you have are mentioned in the text of the paper so that the reader can find them.

DISCUSSION:

- Line 275-279: The authors still need to clarify why this was done, and why it is important. This must be done in the Introduction, and clarified again here.
- Line 280-281: However, at no point is it clear why this information is important to the reader. The authors do mention conceptual reasons for the study in the introduction and sparsely in other sections, but this needs to be consistently done throughout the manuscript.

CONCLUSION

- Finally, improve the conclusion section: it is important to suggest possible future studies.
