# Supplementary material for: The effects of COVID-19 mitigation measures on physical activity (PA) participation among adults in Rwanda: An online cross-sectional survey
Source: PLoS One. 2023 Nov 9;18(11):e0293231. doi: 10.1371/journal.pone.0293231 (PMC10635554; doi:10.1371/journal.pone.0293231)
Supplement: Attachment — Submitted filename: Response to Reviewers.docx [file pone.0293231.s001.docx]

**RESPONSE TO REVIEWERS| Submission ID: PONE-D-22-31575**

**The effects of COVID-19 mitigation measures on physical activity participation among adults in Rwanda.** Nganabashaka JP et al

The research team appreciates the comments provided by the reviewer and the academic editor, as they have significantly contributed to the improvement of this manuscript. The following tables consist of our responses to the comments. The manuscript has also been updated accordingly.

**Table 1: Responses to comments provided by the academic Editor**

| **Comment** | **Response** | **Reference in the manuscript (Page or paragraph (if any amendment)** |
| --- | --- | --- |
| 1. Please ensure that your manuscript meets PLOS ONE's style requirements, including those for file naming. The PLOS ONE style templates can be found at https://journals.plos.org/plosone/s/file?id=wjVg/PLOSOne_formatting_sample_main_body.pdf and https://journals.plos.org/plosone/s/file?id=ba62/PLOSOne_formatting_sample_title_authors_affiliations.pdf. | The manuscript was revised according to the provided guidelines. | All the manuscript |
| 2. Please provide additional details regarding participant consent. | The online survey started with an informed consent statement that provided full description of the study to enable a participant to take an informed and voluntary decision to participate in the study. After reading the informed consent statement, the participant was requested to select “agree” button to access the questionnaire if he/she decided to participate in the study or “disagree” button to end the form if he/she did not want to participate. Participants’ decision (to agree or disagree to participate in the study) was recorded and retrieved from the server for archiving and witness purpose. | See the ethical consideration section page:8-9 |
| 3. We note that you have indicated that data from this study are available upon request. PLOS only allows data to be available upon request if there are legal or ethical restrictions on sharing data publicly. | The 2012 Policy of health sector research in Rwanda requires that researchers desiring to access and use health research databases to consent to the country Ministry of Health, and comply with the “law of access to information” issued by the Rwanda Parliament, to access to research databases. Request for access to raw data used for this study findings should be addressed to the Rwanda National ethics community (RNEC) at [info@rnecrwanda.org](mailto:info@rnecrwanda.org) with a copy to the RNEC chairperson at [jmazarati@gmail.com](mailto:jmazarati@gmail.com)  and to the corresponding author at [nganajp@gmail.com/](mailto:nganajp@gmail.com/) p.nganabashaka.ur.ac.rw. |  |
| 4. Please review your reference list to ensure that it is complete and correct. | Thank you for the critical observation. We found that some references were no longer accessible (example of some government announcements regarding COVID-19 mitigations measures were no longer accessible from the website) and were either removed, replaced or edited accordingly. | All the manuscript |

***Table 2: Responses to comments provided by reviewers***

| **Comment** | **Response** | **Reference in the manuscript (Page or paragraph (if any amendment)** |
| --- | --- | --- |
| 1- I suggest including in the title some information about the type of study (a cross-sectional online survey on COVID-19 or a cross-sectional retrospective study.). | Your input is appreciated, the suggestion executed. | See the title |
| 2. Abstract- Methods: What was the target population for this study? How was the questionnaire disclosed and accessed by the participants? The IPAQ could also be mentioned here as an instrument for the analysis of sedentary lifestyle | Thank you for the comment, the target population was the adult Rwandan aged 18 years and above from the selected sites. The questionnaire link was sent directly to the participants address (on phone through WhatsApp or email) and was provided with instruction and needed assistance by a trained data collector. IPAQ was mentioned in method section: for the sake of meeting journal requirement for 300 words count, these details were put in the method section instead of the abstract. | See Line 110-112  S129-134  See line 55-156 |
| 3- line 100: Please, withdraw 1 “study” (This was a cross-sectional retrospective study.) | Thank you. This is well received and executed. | See line 101 |
| 4- line 112: It was calculated using G*Power 3.1.9.7 calculations. Using G*Power 3.1.9.7 (is repetitive). | Thank you. This is well received and executed. | See line 113-114 |
| 5- line 127-128: This sentence deserves one more reference today. | Well received, the reference was inserted | See line 127 |
| 6- line 152-154: The effect of COVID-19 related mitigations measures on physical activity were measured through the changes in duration spent in different physical activities. (this content shouldn't be here) | The content was removed as per suggestion. Thank you. | See line 154-155 |
| 7- This current study must have followed some guideline (maybe STROBE?) <https://www.strobe-statement.org/checklists/>. It would be very important to include this information. | The STROBE guidelines was followed and was indicated in the manuscript | See line 157-158 |
| 8- Declaration of Helsinki needs to be cited in ethical considerations as well. | The suggestion is executed .Thank you. | See line 160-161 |
| 9- Table 2: include in the legend, the description for the acronym PA and inform the level of significance considered in these analysis. | Thank you. Your suggestion executed | See line 154-155 for significance level and table 2 description |
| 10- line 153, 154, 199, 202, 261, 264, 267, 268, 269, 284, 287, 290, 295, 296, 312, 313, 320, 327, 342,: use PA for physical activity. | Thank you. Your suggestion is executed | See line the corresponding lines in the manuscript |
| 11- Although the authors consider that data collected in different locations with a diverse sample, make the results more robust; I think it is worth noting here the cultural and religious differences that may justify certain findings | Thank you, the suggestion is executed | Line 359-360 |
