# Supplementary material for: The effects of COVID-19 mitigation measures on physical activity (PA) participation among adults in Rwanda: An online cross-sectional survey
Source: PLoS One. 2023 Nov 9;18(11):e0293231. doi: 10.1371/journal.pone.0293231 (PMC10635554; doi:10.1371/journal.pone.0293231)
Supplement: Attachment — Submitted filename: Response to Reviewers.docx [file pone.0293231.s003.docx]

**RESPONSE TO REVIEWERS| Submission ID: PONE-D-22-31575**

**The effects of COVID-19 mitigation measures on physical activity (PA) participation among adults in Rwanda.** Nganabashaka JP et al

The research team appreciates the comments provided by the reviewers and the academic editor. This has significantly contributed to the improvement of this manuscript. We have provided responses to the provided reviewers’ comments and the manuscript has equally been updated accordingly.

**Table 1: Responses to comments provided by the academic Editor**

| **Comment** | **Response** | **Reference in the Revised Manuscript with Track Changes** |
| --- | --- | --- |
| 1. Note that one or more reviewers has recommended that you cite specific previously published works. As always, we recommend that you please review and evaluate the requested works to determine whether they are relevant and should be cited. It is not a requirement to cite these works. We appreciate your attention to this request | Thank you for this, we have carefully read and found that this paper was comparing elite vs the amateurs which is quite different from what this study explored. We opted not to use it for this study. | NA |
| 2. Please review your reference list to ensure that it is complete and correct. If you have cited papers that have been retracted, please include the rationale for doing so in the manuscript text, or remove these references and replace them with relevant current references. Any changes to the reference list should be mentioned in the rebuttal letter that accompanies your revised manuscript. If you need to cite a retracted article, indicate the article’s retracted status in the References list and also include a citation and full reference for the retraction notice | The references list has been checked. No cited paper has been retracted. | All the manuscript |
| **Response to comments provided by reviewer #2** |  |  |
| **Comment** | **Response** | **Reference in the Revised Manuscript with Track Changes** |
| Work more on abstract. Here, you can see recommendations: Overall, the abstract provides a clear and concise summary of the study's objectives, methods, and results. However, there are some areas where the abstract could be improved: The abstract does not clearly state the type of physical activity that was assessed. The study evaluated the variation in time spent doing different PA types, but the abstract does not provide any information on what those types were. The abstract could benefit from more specific information on the study population. While the abstract mentions that 1136 adults above 18 years old completed the questionnaire, it does not provide any information on their demographic characteristics or how they were selected.  it may be useful to highlight the importance of this research, even four years after the emergence of COVID-19. While the study's findings provide valuable insights into how COVID-19 mitigation measures impact physical activity participation in Rwanda, the implications of these findings extend beyond the current pandemic. use the following references for that: Taheri, Morteza, et al. "Effects of home confinement on physical activity, nutrition, and sleep quality during the COVID-19 outbreak in amateur and elite athletes." Frontiers in nutrition 10 (2023): 1143340. | Thank you very much for the very important comments. We have partially responded to your comments. We could not provide detailed information in the abstract because of the words limit. As you can see from this link: <https://journals.plos.org/plosone/s/submission-guidelines#loc-abstract>, the abstract should not exceeded 300 words.  Thank so much for the reference paper; we have added a sentence on the need for this kind of studies even after years of the pandemic. | See the abstract  See line 101-106 on page 6 |
| **Response to comments provided by reviewer #3** | | |
| **Comment** | **Response** | **Reference in the Revised Manuscript with Track Changes** |
| Overall this is an interesting study which could have much interest, however, I do have some concerns as follows: | Thank you very much, your comments are greatly appreciated. |  |
| In the list of authors, you have listed No 2 Pettenfer School of Public health, but none of the authors are cited at that institution. Please clarify | Thank you very much, when we first drafted this manuscript, there was a co-author (Kerstin Sell) from Pettenfer School of Public health but as we finalize the manuscript, she was busy and could not contributed enough to qualify as a co-author. She was acknowledged for her effort. It was our error to keep it in the list of affiliation. | The Pettenfer School of Public health was removed from the list of affiliation |
| You have stated a very low number of Covid cases in Rwanda, which is due to low amounts of testing. It would be good to give this detail as many readers will not realise why the number of cases are so low. This has been supported by the World Health Organisation who states that the numbers are low due to low testing. | Thank you for these comments. It was noted that one of the reason for the low COVID-19 cases was low testing rate. However, we could not attributed this to the case of Rwanda with no tangible evidence. | NA |
| In the abstract, it would be good to mention that statistical analysis was carried out rather than just listing percentage change. | Thank you for the comments. As you can see from this link: <https://journals.plos.org/plosone/s/submission-guidelines#loc-abstract>, Plos one guidelines, request that we provide key results in the abstract which is why we have included some key findings. | NA |
| Line 65 upon not up on | Thank you, your comment is executed | Line 71 on page 5 |
| Line 69 what is NCD alliance? | Thank you, your comment is well received, NCD alliance was briefly explained. | Line 75-81 on page 5 |
| Line 70 rebroadcast not rebroadcasted | Thank you the word is replaced | Line 80 on page 5 |
| Line 71 should be 'activity program is believed' not 'are believed' | Thank you, this is well appreciated and executed | Line 84 on page 5 |
| Line 72 mainaining not maintain | Thank you the word is replaced | Line 84 on page 5 |
| Line 146 – serious reservations about this time period as many counties were still in and out of lockdown, therefore had people gone back to pre covid situation? Were government backed PA activities back up and running? if not this will scew your results as people were not completed out of lockdown | Thank you very much. As explained in method section 118-121, After 4^th^ May 2020 the outdoor PA were resumed with only individual PA. | NA |
| Line 167 was 29 years not with 29 years | Thank you, this is well appreciated and executed | Line 188 on page 10 |
| Line 166 put 71.4% in brackets | Thank you, this is well appreciated and executed | Line 187 on page 9 |
| Is it a bias that 58.76% have university education when statistics show that in 2019 only 12.8% of Rwandan population had higher education? | Thank you for your comments. Our thoughts n this is that the study targets a specific people (those participating in PA at the selected sites) and were selected conveniently therefore the results are not going to be country representative. However, they are still useful for interventions design and policy making. | NA |
| Line 175 should be latter not later | Thank you, this is well appreciated and executed | Line 197 on page 11 and throughout the whole manuscript |
| Line 191 what is meant by 'walking as part of the work'? This seems to have slightly increased during total lockdown when hardly anyone was at work. This needs explaining. | Thank you for the comment. Walking as part of the work for this study was defined as time spent doing usual work such as paid jobs, farming, volunteering works, course work and any other unpaid work that is outside of participant’s home. As explained in the background page 4 line 67-68, some essential services were allowed; however, we have not enough evidence to support the causal relationship with this finding. Again, this was a self-reported data that we have little control on desirability bias. Contradicting findings are quite common in this kind of studies (Impact of COVID-19 on physical activity: A rapid review: **ttps://www.ncbi.nlm.nih.gov/pmc/articles/PMC8979477/).** We acknowledged this as a limitation for this study. | Line 368-369 on page 18 |
| Line 212 again it doesn’t make sense how bike riding only reduced by 5 minutes a day if you were locked down | Thank you for the comment. As explained in previous comments, this was a self-reported data that we have little control on desirability bias. We put this as a limitation for this study. Additionally, bikes and motorcycles were the most used means for transporting essential commodities during Covid-19. This was not discussed because the findings was not statistically significant | NA |
| What is the difference between daily sitting time and sedentary sitting time line 228? Please explain | Sedentary time include both sitting and sleeping time. It was an error to write sedentary sitting time and was corrected. | Line 256 on page 13 |
| Line 240 there is a repeat of half a sentence about the Bonferroni test, please remove | Thank you, this is well appreciated and executed | Line 269-270 on page 14 |
| Paragraph starting line 257 is hard to understand and the whole paragraph needs re-wording. | Thank you, this is well appreciated and rewording was executed | Line 256-261 on page 13-14 |
| Line 263 explain why old people present barriers to PA, especially when you are talking of people age 35y and over, this isn’t particularly old | Thank you, this intended to explain the youth dominance of this study’s participants. It was well articulated in the manuscript. | Line 291-292 on page 15 |
| Line 274 should be before the emergence of covid 19 not the before | Thank you, this is well appreciated and executed | Line 274 on page 14 and across the manuscript |
| Line 275 where were these other studies that are referred to? in Rwanda or other countries? | Thank you. The referred studies (32,33) were conducted from Ireland and Australia respectively and was mentioned in the manuscript | Ligne 294-295 on page 15 |
| Line 308 ; other countries allowed to go outside and exercise during lockdown, but not Rwanda, so how did they bike ride during lockdown? You have said that this only decreased by 5% during lockdown, which doesn’t make sense. | Thank you for the comment, some essential services were allowed during lockdown. Moreover, as stated in manuscript line 81-82 on page 5 government has intermittently allowed people to exercise within their respective villages’ boundaries; Again, this was a self-reported data that we have little control on desirability bias. We put this as a limitation for this study. | Line 368-369 on page 18 |
| Line 323 not resume but resumption | Thank you, this is well appreciated and executed | Line 350 on page 18 |
| Line 341 this section doesn’t make sense. You have said that sitting time increased in your results but now say that it did not increase during lockdown. | Thank you so much for the comment. The section was comparing the found sitting time with Worldwide self-reported sitting and National Sleep Foundation’s updated sleep duration with the found sleep time but with your comment, we realized that this comparison is confusing and does not add value. We have remove these comparison | Line 363-368 on page 18 |
| Needs more discussion on limitations of the study eg that is was retrospective and the bias of males vs females and age | Thank you. The limitations were mentioned in the discussion section. | Line 371-373 on page 18 |
| Does the conclusion contradict earlier statements about home based PA programmes as you are saying they are efficient but earlier you have said that people get bored of them? | Thank you, In the discussion section, specifically lines 339-344 on page 17, we mentioned that home-based physical activity was not strongly promoted and explored during the lockdown. However, in the conclusion, we recommend giving it more attention and exploring it fully. To avoid any confusion, we have rephrased the sentences in the discussion section. | Line 339-344 on page17 |
| Some of the english is difficult to understand and I would suggest that you get someone with english as a first language to proof read this. | Thank you very much, proof reading was done by the English fluent co-author. | NA |
| **Response to comments provided by reviewer #4** | | |
| **Comment** | **Response** | **Reference in the Revised Manuscript with Track Changes** |
| Thank you for your submitted manuscript entitled, PONE-D-23-13290 ‘’The Effects of COVID-19 Mitigation Measures on Physical Activity Participation among Adults in Rwanda: an Online Cross-sectional Survey’’ | You are most welcome and your comments are greatly appreciated. |  |
| ABSTRACT: • In the Methods provides more information about the PA and sedentary time assessment used in the study. | Thank you very much. As you can see from this link: <https://journals.plos.org/plosone/s/submission-guidelines#loc-abstract>, the abstract should not exceeded 300 words. We could not provide detailed information in the abstract. |  |
| • How you determined the ‘’self-administered questionnaire’’. | Thank you so much your comments allowed us to recognize that the questionnaire was not fully self-administered since some assistance was virtually provided to some participants especially those who were not familiar with online data collection. We removed the self-administered to assisted questionnaire. | Line 30 on page 2 |
| • Could be a relevant conclusion for the present study. However, you note in the conclusion that ‘’COVID-mitigation measures have significantly reduced PA participation and increased sedentary time’’.This statement is not a new discovery. Although, what is the new discovery? | Thank you for this pertinent comment. This study has particularly explored the practice of the PA in all aspect of life including transportation, recreation, housework, etc, which some of them were not well explored but rather useful in difficult time such as during lockdown. It explored how the pandemic affected the population’s sedentary lifestyle. It is a great inputs and reminder of the intervention designers and policy makers to extend their effort recreational outdoor PA and normalize other types of PA such home based, indoor, work related, etc. This has been put in the manuscript. | Conclusion sections page 3 and 19 |
| INTRODUCTION: • Line 97-108: The development of the introduction needs to be more hypotheses driven and develop the questions leading up to the section in the methods section. In this section, there is no new information in the paper this is reported in many published papers, it is necessary to remake and reinforce this part by other evidence, information and bibliographic references. Further, the part on hypothesis/-es development needs revision. A more conclusive use of appropriate literature can help here to clearly state the purpose of the study in order to develop hypotheses (or null hypotheses, whichever way is preferred). In turn, this will serve as the paper's framework as it will define the importance of findings, i.e. which results are being presented. In its present form, the paper states one quite unspecific hypothesis, therefore, results presented seem random which also influences the discussion. | Thank you very much for the comments that made the manuscript much better. The introduction was revised accordingly. | Introduction section |
| METHOD • The procedure is rigorous and well described and the statistical analysis is correct. | Thank you very much |  |
| RESULTS: What about the measure of data reliability in the study? | The statistician checked the data submission progress, completeness, validity and accuracy by reviewed each submitted form. He daily provides feedback to the data collectors following up the participants to make sure that any raised concern is solved. Duplicates were cleaned and removed before data analysis. | Line 169-171 on page 9 |
| The tables are poorly presented, need formatting. Take time to check all formatting and make sure that all of the tables you have are mentioned in the text of the paper so that the reader can find them. | Thank you. All tables were reformatted (where needed) and check for coherence in text | See table 1,2 and 3 and their narrative |
| DISCUSSION: Line 275-279: The authors still need to clarify why this was done, and why it is important. This must be done in the Introduction, and clarified again here | Thank you the discussed was revised as well as the introduction. | Introduction section line 91-125 on page 5-6  Line 286-290 of the discussion on page 15 |
| Line 280-281: However, at no point is it clear why this information is important to the reader. The authors do mention conceptual reasons for the study in the introduction and sparsely in other sections, but this needs to be consistently done throughout the manuscript. | Thank you so much for the good comment. We have introduced the uniqueness of the study and its importance to the audience | Line on page 297-402 on page 19-20. |
| CONCLUSION Finally, improve the conclusion section: it is important to suggest possible future studies. | Thank you this is well executed | Conclusion section page 19 |
